# Correction: Cai et al. Roles of Three *FgPel* Genes in the Development and Pathogenicity Regulation of *Fusarium graminearum*. *J. Fungi* 2024, *10*, 666

**DOI:** 10.3390/jof11010010

**Published:** 2024-12-27

**Authors:** Lu Cai, Xiao Xu, Ye Dong, Yingying Jin, Younes M. Rashad, Dongfang Ma, Aiguo Gu

**Affiliations:** 1 Key Laboratory of Sustainable Crop Production in the Middle Reaches of the Yangtze River, College of Agriculture, Yangtze University, Jingzhou 434025, China; 2023710834@yangtzeu.edu.cn (L.C.); xuxiao0913@foxmail.com (X.X.); 2021720806@yangtzeu.edu.cn (Y.D.); 202072787@yangtzeu.edu.cn (Y.J.); 2 Jiangsu Academy of Agricultural Sciences, Jiangsu Coastal Area Institute of Agricultural Sciences, Yancheng 224000, China; 3 Plant Protection and Biomolecular Diagnosis Department, Arid Lands Cultivation Research Institute (ALCRI), City of Scientific Research and Technological Applications (SRTA-City), New Borg El-Arab 21934, Egypt; younesrashad@yahoo.com; 4 Jiangsu Product Quality Testing & Inspection Institute, Nanjing 210007, China; nj180618@163.com

## Text Correction

In the original article [1], in Section 2.2 of Materials and Methods, an extraneous passage was included that is unrelated to the experimental methods and has therefore been removed. Additionally, in this study, the phylogenetic tree was constructed using the neighbor-joining (NJ) method, which was implemented in MEGA 11.0 software, rather than the MUSCLE tool. The corrected paragraph is as follows:

The protein sequences of the three genes (GenBank registry numbers: NC_026475.1, NC_026474.1, and NC_026476.1) were downloaded from the NCBI database and subsequently visualized. The selected protein sequences were analyzed using the online software SMART 9.0 (http://smart.embl-heidelberg.de (accessed on 23 February 2024)) and Pfam 35.0 (http://pfam.xfam.org/search (accessed on 23 February 2024)) to identify their structural domains, which were then visualized using IBS 2.0. The predicted amino acid sequences were compared and used to construct the phylogenetic tree using the neighbor-joining (NJ) method, which was implemented in MEGA 11.0 software [28,29].

In Section 3.3 of the Results in the original article, there was an error in the subheading. The correct subheading should be:

*3.3. The Generation of Three FgPel Gene Deletion Mutants.*

## Removal of Citation

The removal of the text described above also requires the removal of the following citations from the original text:

29. Edgar, R.C. MUSCLE: Multiple sequence alignment with high accuracy and high throughput. *Nucleic Acids Res*. **2004**, *32*, 1792–1797.

All other citations should be renumbered to reflect the removal of the above citations.

The authors point out that the scientific conclusions are unaffected by these changes. All co-authors agree with the content of this correction and apologize to the readers for any inconvenience caused by this error.
