# Supplementary material for: Roles of Three FgPel Genes in the Development and Pathogenicity Regulation of Fusarium graminearum
Source: J Fungi (Basel). 2024 Sep 24;10(10):666. doi: 10.3390/jof10100666 (PMC11508199; doi:10.3390/jof10100666)
Supplement: Supplementary file 1 [file jof-10-00666-s001.zip › Table S1.pdf]

Supplementary Table S1. Primers used in this study.

**Name**	**Sequence（5′-3′）**
FgPel1-1F	GGGAGGATACAACAACAAGGG
FgPel1-2R	TTGACCTCCACTAGCTCCAGCCAAGCCAGGATTCTCGGATTCTTCGG
FgPel1-3F	GAATAGAGTAGATGCCGACCGCGGGTTCAACCAATCCCAAATCTTACC
FgPel1-4R	GGCACTTGTTCAAACCCAG
FgPel1-5F	AGCAAGGCTGGAACGAGTG
FgPel1-6R	GACCTGGCAATAGGCAATCC
FgPel1-7F	GCTTCTGGGACTTCAACAACG
FgPel1-8R	AGCCCAGAAACCAAAGCATC
FgPel2-1F	AGCCGCATCGTATCGTAGTG
FgPel2-2R	TTGACCTCCACTAGCTCCAGCCAAGCCGCGATACTCCCATCCCTCAG
FgPel2-3F	GAATAGAGTAGATGCCGACCGCGGGTTAGTTTTTCTCAGCAGCAAGGAC
FgPel2-4R	TTTCCCGATTCAGTTTCCATTAC
FgPel2-5F	ACCAGCGAGCCCATTGAG
FgPel2-6R	CAAGCGTTGGAGATGGTGC
FgPel2-7F	CAGAGAGATAGGAACTGGCTTTG
FgPel2-8R	TCTTACACTGACAAGGGTCTGCTA
FgPel3-1F	CTGAGTAGTCGGGTAGTCTGAAGC
FgPel3-2R	TTGACCTCCACTAGCTCCAGCCAAGCCGAGCAAACAGCACTTGAGGGA
FgPel3-3F	GAATAGAGTAGATGCCGACCGCGGGTTAATCCGCAACTCCACGACTG
FgPel3-4R	CGGTCCGTAGTTTCTAAGTGTCTG
FgPel3-5F	CAACGCCATCATTGAGGGTA
FgPel3-6R	CTCGTACTGGGAATCGTAATCG
FgPel3-7F	TCATCAATACCAATGCCAAACC
FgPel3-8R	AACCCAAAGAAGACGGACGA
HYG/F	GGCTTGGCTGGAGCTAGTGGAGGTCAA
HY/R	GTATTGACCGATTCCTTGCGGTCCGAA
YG/F	GATGTAGGAGGGCGTGGATATGTCCT
HYG/R	AACCCGCGGTCGGCATCTACTCTATTC
H852	TTCCTCCCTTTATTTCAGATTCAA
H850	ATGTTGGCGACCTCGTATTGG
H855R	GCTGATCTGACCAGTTGC
H855F	GTCGATGCGACGCAATCGT

FgPel11-q1F	CAAGCAAGTTGTCGTTCCCG
FgPel11-q2R	CTCCGACATGAAGCCGGTAT
FgPel12-q1F	GTTCTCTGCGGCATCAACAC
FgPel12-q2R	CTTGCAAGAAGTGCCATCGG
FgPel13-q1F	CAAAGACTGGCCCTGGATGT
FgPel13-q2R	CAACAGGAAGAGGCTCAGGG
