# Supplementary material for: Roles of Three FgPel Genes in the Development and Pathogenicity Regulation of Fusarium graminearum
Source: J Fungi (Basel). 2024 Sep 24;10(10):666. doi: 10.3390/jof10100666 (PMC11508199; doi:10.3390/jof10100666)
Supplement: Supplementary file 1 [file jof-10-00666-s001.zip › Table S2 .pdf]

Supplementary Table S2. Structure quality estimation of predicted pectin lyase prote in models of *Fusarium* species.

No.	Protein	Favored region	Allowed region	Outlier region
1	*Fgpel*1	82.3%	16.8%	0.3%
2	*Fgpel*2	78.9%	14.4%	3.5%
3	*Fgpel*3	68.7%	16.3%	5.5%
