# Supplementary material for: Roles of Three FgPel Genes in the Development and Pathogenicity Regulation of Fusarium graminearum
Source: J Fungi (Basel). 2024 Sep 24;10(10):666. doi: 10.3390/jof10100666 (PMC11508199; doi:10.3390/jof10100666)
Supplement: Supplementary file 1 [file jof-10-00666-s001.zip › Table S3 .pdf]

Supplementary Table S3 Structure quality estimation of predicted pectin lyase prote in models of *Fusarium* species.

No.	Protein	ERRAT	Verify 3D (%)
1	*Fgpel*1	84.900	92.53%
2	*Fgpel*2	80.083	70.7%
3	*Fgpel*3	81.108	63.97%
