# Supplementary figures and images for: Roles of Three FgPel Genes in the Development and Pathogenicity Regulation of Fusarium graminearum
Source: J Fungi (Basel). 2024 Sep 24;10(10):666. doi: 10.3390/jof10100666 (PMC11508199; doi:10.3390/jof10100666)

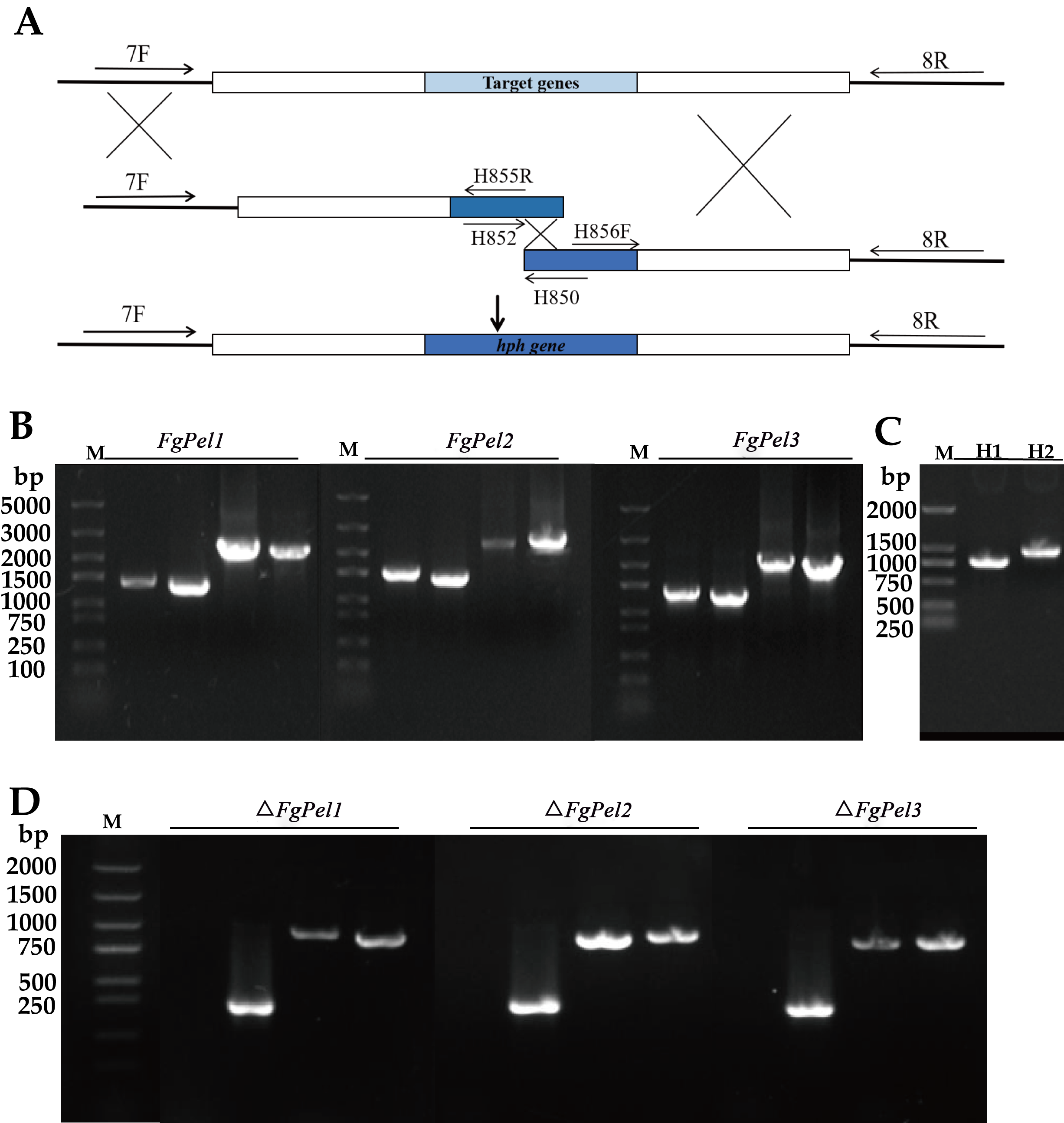

Supplement: Supplementary file 1 [file jof-10-00666-s001.zip › Figure S1.tif]

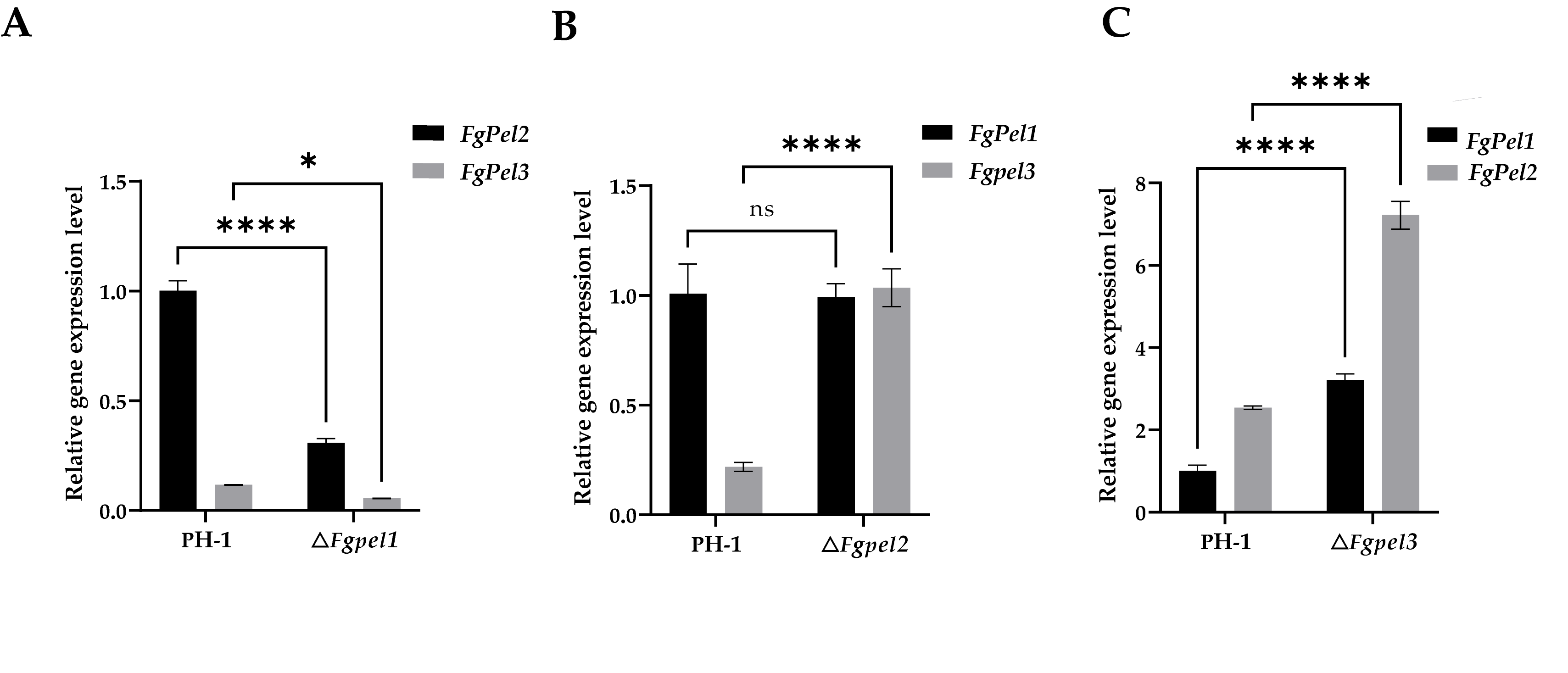

Supplement: Supplementary file 1 [file jof-10-00666-s001.zip › Figure S2.tif]

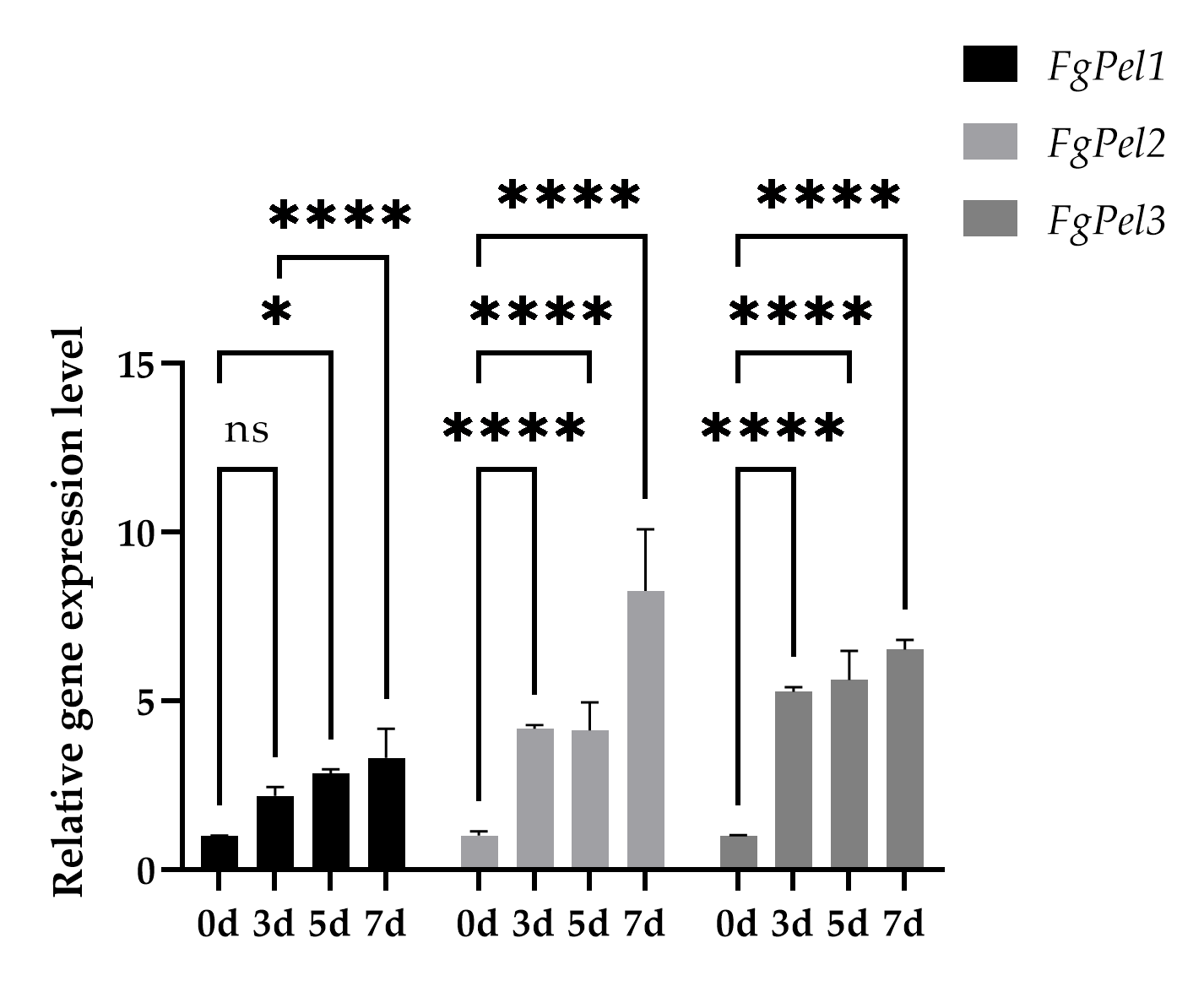

Supplement: Supplementary file 1 [file jof-10-00666-s001.zip › Figure S3.tif]

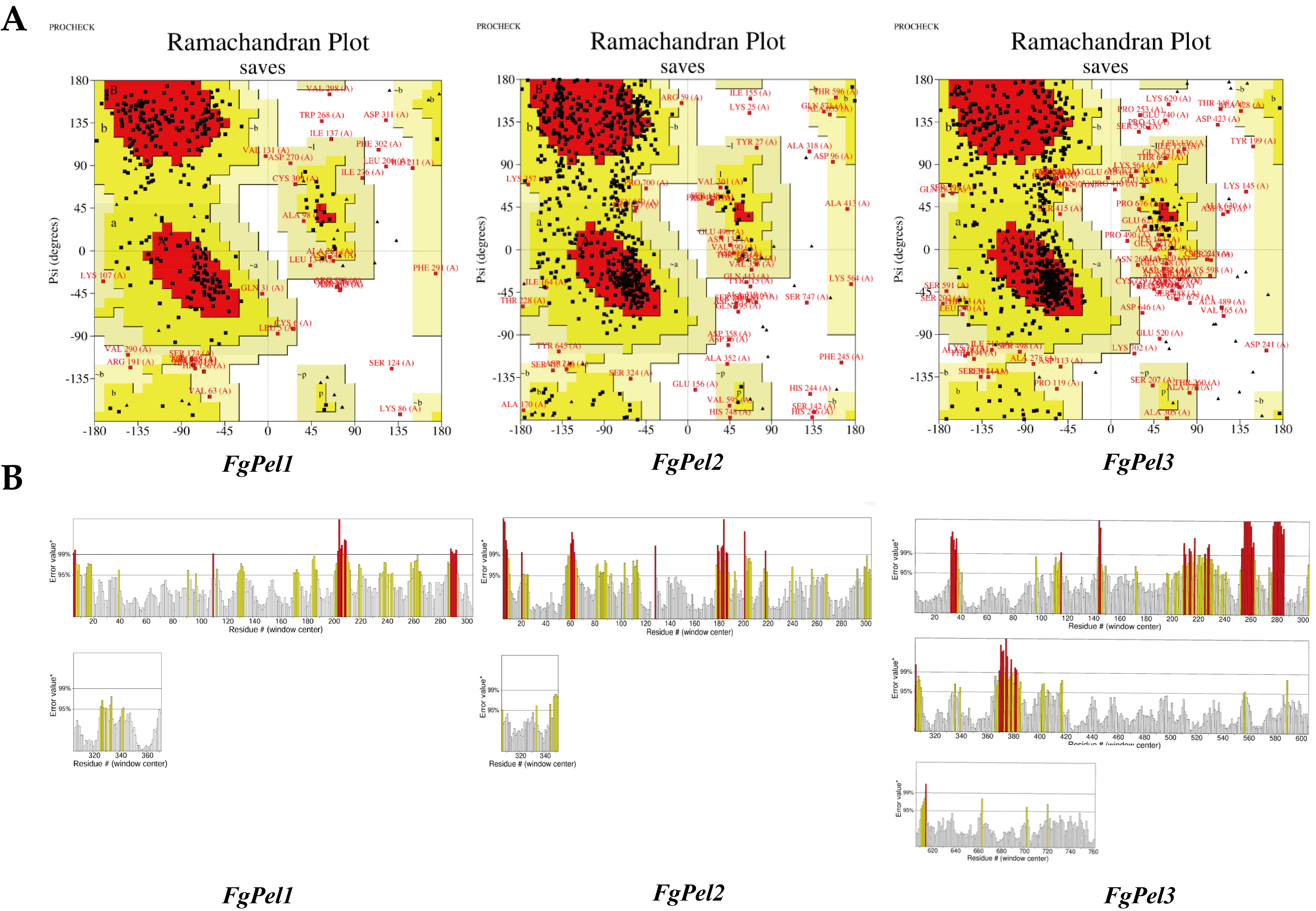

Supplement: Supplementary file 1 [file jof-10-00666-s001.zip › Figure S4.tif]

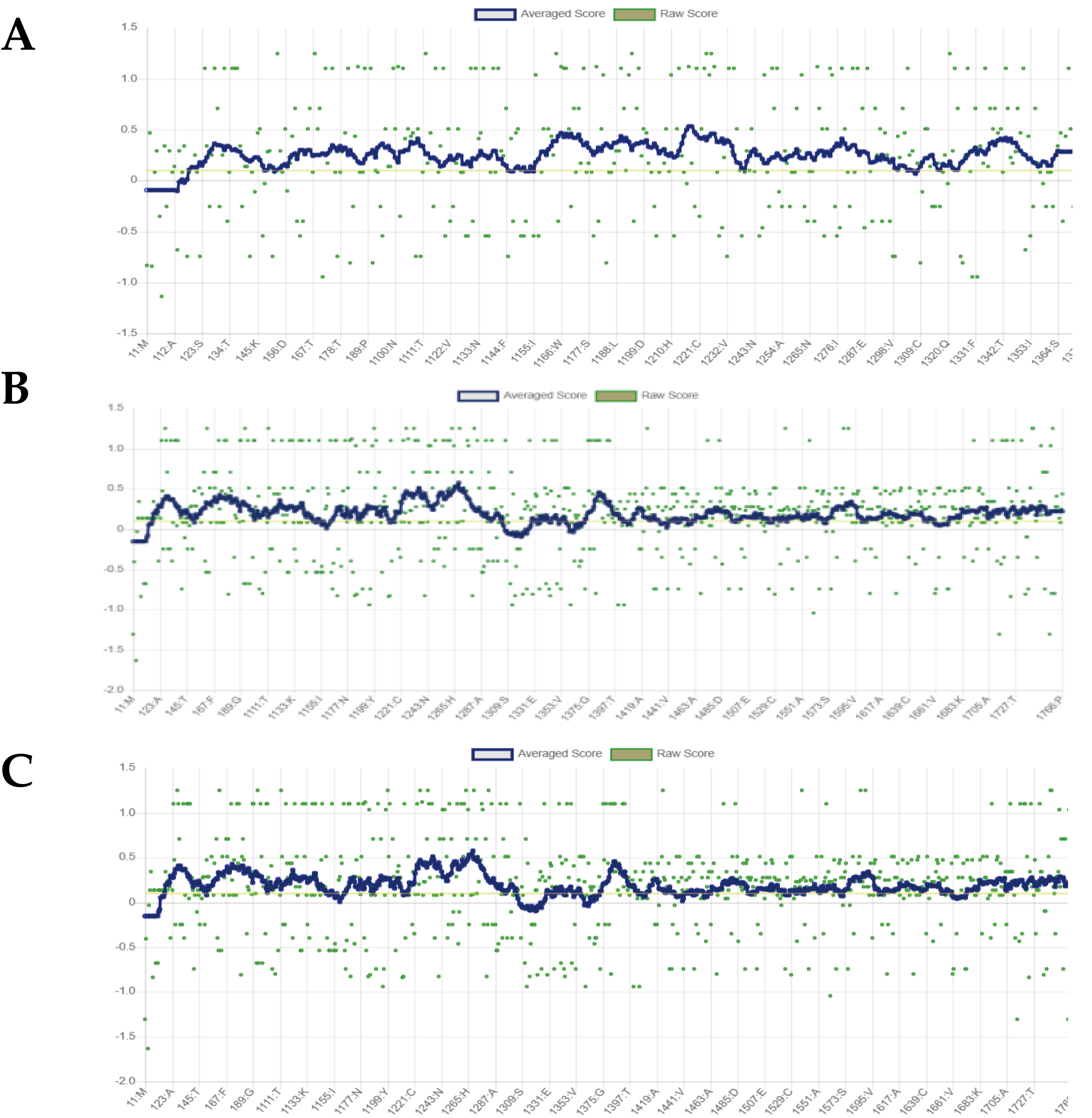

Supplement: Supplementary file 1 [file jof-10-00666-s001.zip › Figure S5.tif]
